# Whole-genome characteristics of isolates from recurrent *Clostridioides difficile* infections

**DOI:** 10.1128/spectrum.04087-25

**Published:** 2026-03-27

**Authors:** Lisi Zheng, Tao Lv, Xiao Xu, Hanhan Bao, Ping Shen, Danhua Zhu, Yunbo Chen

**Affiliations:** 1 State Key Laboratory for Diagnosis and Treatment of Infectious Diseases, National Clinical Research Center for Infectious Diseases, China-Singapore Belt and Road Joint Laboratory on Infection Research and Drug Development, National Medical Center for Infectious Diseases, Collaborative Innovation Center for Diagnosis and Treatment of Infectious Diseases, The First Affiliated Hospital, Zhejiang University School of Medicine 26441, Hangzhou, Zhejiang, People’s Republic of China; 2 School of Laboratory Medicine and Life Sciences, Wenzhou Medical University 214181 , Wenzhou, Zhejiang, People’s Republic of China; 3 Jinan Microecological Biomedicine Shandong Laboratory 661980 , Jinan, Shandong, People’s Republic of China; Johns Hopkins University, Baltimore, Maryland, USA

**Keywords:** *Clostridioides difficile*, recurrent *Clostridioides difficile *infection, relapse, reinfection, whole-genome sequencing

## Abstract

*Clostridioides difficile* infection (CDI) imposes considerable medical and economic burdens on patients and healthcare systems due to its high recurrence rate and treatment refractoriness. Recurrent CDI (rCDI) may result from a relapse of the same strain or reinfection by a different strain. However, the genetic and evolutionary features of strains causing rCDI remain poorly understood. Therefore, in this study, CDI strains were collected from patients with ≥2 clinically defined infections. Through antimicrobial susceptibility testing and whole-genome sequencing (WGS), we investigated the correlation between relapse and reinfection strains. WGS revealed that 40 of 82 patients with CDI (48.8%) relapsed with the original strain, including 6 cases exceeding the 8-week recurrence interval definition. The recurrence interval was significantly shorter for relapse cases than for reinfections. Moreover, the relapse rate was significantly higher in hospital-associated (HA-CDI) cases than in community-associated (CA-CDI) cases (*P* < 0.05). Notably, strains isolated from relapse cases showed a trend toward higher resistance rates to clindamycin, imipenem, moxifloxacin, and tigecycline than those from reinfection cases. No significant genomic differences, including resistance genes, virulence genes, and transposons, were observed between relapse and reinfection strains. Core genome evolutionary analyses indicated that both types were predominantly under purifying selection, with only a few genes related to transcriptional regulation and energy metabolism under positive selection. The findings indicate that relapse and reinfection strains share similar evolutionary drivers, suggesting that recurrence may be more closely associated with other factors, such as host gut microbiota or gene expression regulation.

**IMPORTANCE:**

*Clostridioides difficile* infection (CDI) poses a major challenge in healthcare settings due to its high recurrence rate. Accurate differentiation between relapse of the original strain and reinfection with a new strain is crucial for effective management of recurrent *Clostridioides difficile* infection (rCDI). This study combined whole-genome sequencing (WGS) and antimicrobial susceptibility testing to thoroughly examine the genomic characteristics of isolates from rCDI patients at our hospital, aiming to identify potential genomic features and key factors contributing to rCDI. Core genome analysis revealed highly consistent evolutionary trajectories between recurrent and reinfection strains, with no significant differences in their genetic lineages. This finding suggests that recurrence tendencies may be influenced more by host-specific factors or regulatory adaptations. These insights offer a new direction for developing targeted management strategies for rCDI.

## INTRODUCTION

*Clostridioides difficile* (*C. difficile*), an anaerobic, spore-forming Gram-positive bacterium, is one of the most common pathogens causing antibiotic-associated diarrhea (1). *C. difficile* primarily causes *C. difficile* infection (CDI) through two virulence factors (toxin A and toxin B), with clinical manifestations including diarrhea, fever, and abdominal pain. Severe cases may lead to pseudomembranous colitis, toxic megacolon, or even death (1). Currently, stringent control of nosocomial infections, the emergence of novel treatment modalities, and the incidence and mortality rates of CDI have decreased through enhanced clinical awareness. However, approximately one-third of patients still experience recurrent CDI (rCDI) after initial treatment (2, 3). Recurrent CDI (rCDI) is typically defined as a new diagnosis of CDI occurring within 8 weeks of recovery from the previous CDI episode (4). Current clinical practice guidelines define CDI diagnosed more than 8 weeks after recovery as a new infection rather than a recurrence (4, 5). According to previous studies, patients who have experienced one recurrence face a markedly increased risk of subsequent rCDI, with recurrence rates rising to 45%–65% (2). Risk factors associated with rCDI include advanced age, severe underlying medical conditions, immunocompromised status, antibiotic use, gastric acid suppression, and infection with highly virulent strains (6). rCDI increases the medical and psychological burden on patients, prolongs hospital stay, and leads to increased healthcare costs (7, 8). This makes rCDI a major challenge in healthcare settings.

rCDI can result from relapse of the same strain and reinfection by a different strain (9). Its occurrence relates not only to host immune status and the intestinal microenvironment but also to the genetic characteristics of the infecting strain itself. For example, epidemic *C. difficile* strains NAP1/BI/027 produce not only toxin A and toxin B but also a binary toxin (CDT). Compared to other strains, it exhibits stronger adhesion and colonization capabilities as well as greater drug resistance, leading to higher recurrence rates in patients (10). Furthermore, Kulecka et al.’s study on rCDI strains revealed that genes associated with carbon metabolism and oxidative phosphorylation also increase the risk of recurrence (11). Therefore, understanding the genomic characteristics and resistance profiles of strains from a genetic perspective is essential for elucidating the recurrence mechanisms of rCDI recurrence mechanisms.

Different types of recurrence caused by relapse and reinfection require distinct clinical treatment and management strategies (4). Currently, traditional molecular typing methods such as PCR-ribotyping cannot provide adequate resolution to distinguish between relapse and reinfection (12, 13). Accurately differentiating infection types in rCDI is crucial. With the advancement of molecular biotechnology, WGS offers marked advantages in identifying highly similar strains, elucidating pathogenic resistance mechanisms, and tracing genetic evolution (9). However, at present, the lack of characteristic research on rCDI strains in mainland China has limited the implementation and development of precise prevention and treatment strategies in clinical practice. Therefore, in this study, we aimed to analyze the genomic characteristics of relapse and reinfection strains using WGS technology, thereby elucidating the genetic and evolutionary characteristics of rCDI. This will provide new insights for subsequent research on the mechanisms of recurrence and the design of rational treatment and prevention measures.

## MATERIALS AND METHODS

### *C. difficile* isolates and the collection of patient clinical data

This study was conducted at the First Affiliated Hospital of Zhejiang University School of Medicine. Fecal samples of diarrheal patients who underwent *C. difficile* testing from 2013 to 2024 were collected. The tests were conducted using *C. difficile* Quick Chek Complete (TechLab, USA) and Vidas *C. difficile* GDH and toxin A/B (Biomérieux, France) test kits, and the procedures were strictly performed per the manufacturer’s instructions. *C. difficile* was isolated and identified from fecal samples that were positive for both GDH and A/B toxin tests. The method is as follows: Fecal samples were cultured on the selective medium cycloserine-cefoxitin-fructose agar (CCFA-TA; Oxoid), incubated anoxically at 37°C for 48 h, as described earlier (14). Colonies that are light yellow have irregular edges, rough surfaces, and special odors were selected for identification and preservation by Matrix-assisted laser desorption/ionization-time of flight mass spectrometry (MALDI-TOF MS, Bruker Daltonik GmbH, Germany). Patients who met the definition of recurrent or new *C. difficile* infection were included, and the relevant clinical information of these patients was collected.

### Definitions

Community-associated CDI (CA-CDI): CDI occurring within 48 h of admission and without a history of hospitalization within 12 weeks prior to admission, or occurring 12 weeks after discharge from a healthcare facility (15).

Healthcare-associated CDI (HA-CDI): CDI occurring in hospitalized patients after 48 h of admission, and CDI occurring within 4 weeks after discharge from a healthcare facility (16).

### WGS and data analysis

Bacterial genomic DNA was extracted using the DNeasy PowerSoil Pro Kit (Qiagen, Germany) according to the manufacturer’s instructions. WGS was performed using libraries on an Illumina NovaSeq 6000 (Illumina, San Diego, CA, USA) instrument. Raw data underwent quality control using FastQC v.0.11.5 (https://www.bioinformatics.babraham.ac.uk/projects/fastqc/), followed by adapter trimming with Trimmomatic v.0.40. Subsequently, the trimmed genomic sequences were reassembled using SPAdes v.3.6 software and annotated using Prokka (17). Genotypes of all *C. difficile* strains were determined based on DNA sequencing data using the PubMLST sequence query page (https://pubmlst.org).

### Core-genome single-nucleotide polymorphism analysis

Construct a maximum likelihood tree (MEGA11) based on core genome alignments obtained from Roary, using 1,000 bootstrap replicates, and visualize it via the interactive PhyloTree web server. Meanwhile, recombination was identified and masked using Gubbins (18), and pairwise single nucleotide polymorphism (SNP) distances within the core genome were calculated from the resulting alignment using snp-dists (https://github.com/tseemann/snp-dists). Based on prior evidence showing that transmitted isolates differed by 0–2 single-nucleotide variants (SNVs) over sampling intervals of 124–364 days (19), a threshold of ≤2 SNPs was applied to infer potential transmission events in this study.

### Identification of antimicrobial resistance genes, virulence genes, and mobile genetic elements

AMR genes were detected using the Comprehensive Antimicrobial Resistance Database (CARD) (https://card.mcmaster.ca). Additionally, virulence genes were identified based on the local Virulence Factor Database (VFDB) (https://www.mgc.ac.cn/VFs/). Mutational analysis utilized Basic Local Alignment Search (BLAST) to extract *gyrA* sequences. MGEs were identified using Vrprofile2 (https://tool2-mml.sjtu.edu.cn/VRprofile/VRprofile.php).

### Pangenome-wide association study and clusters of orthologous groups of proteins analysis

To investigate genomic characteristics during evolutionary change, a pan-genome-wide association analysis was conducted using genes extracted from the pan-genome of all isolates by Roary. Genes showing significant differences between the two groups were identified using Scoary software. Significance was verified using the Kruskal–Wallis test with Bonferroni correction. Gene data from all isolates were extracted using a proprietary Python script and uploaded to the eggNOG database to investigate gene functions (20).

### Antimicrobial susceptibility testing

Strain recovery was performed on Columbia blood agar plates. Brucella agar supplemented with chlorheme and vitamin K1 was prepared. All strains underwent antimicrobial susceptibility testing using the agar dilution method as per the Clinical and Laboratory Standards Institute (CLSI) guidelines. ATCC700057 was used as the quality control strain for the drug sensitivity test to determine the minimum inhibitory concentration (MIC) of 13 antimicrobial agents, including vancomycin, metronidazole, clindamycin, erythromycin, imipenem, moxifloxacin, tetracycline, linezolid, rifampin, tigecycline, fidaxomicin, ceftriaxone, and chloramphenicol. Antibiotic breakpoints primarily follow CLSI guidelines, except for vancomycin (>2 mg/L) and fidaxomicin (>0.5 mg/L), which adopt the epidemiological cutoff values (ECOFFs) from the European Committee for Antimicrobial Susceptibility Testing (EUCAST). Tigecycline (≥16 mg/L) follows the U.S. Food and Drug Administration standard. The breakpoint for linezolid is set at 4 mg/L. Isolates with MICs >32 mg/L are designated as resistant strains as the rifampin breakpoint is not clearly defined.

### Statistical analysis

Data were analyzed using SPSS 26.0 statistical software. Statistical analysis employed the Wilcoxon rank-sum test, Chi-square test, or Fisher’s exact test. Differences were considered statistically significant at *P* < 0.05. The area under the receiver operating characteristic curve (ROC area under the ROC curve [AUC]) was calculated to evaluate the diagnostic discriminatory power for distinguishing between relapse and reinfection. Youden’s index was used to determine the optimal cutoff point for sensitivity and specificity, and the Hosmer–Lemeshow goodness-of-fit test was applied for validation.

## RESULTS

### Cohort characteristics

In total, 3,418 patients were diagnosed with CDI from 2013 to 2023, of whom 375 (11.0%) were diagnosed with recurrent or new infection. After isolation and identification of *C. difficile*, only 82 patients had strains before and after infection, and a total of 167 toxigenic *C. difficile* strains were obtained.

**Fig 1:**
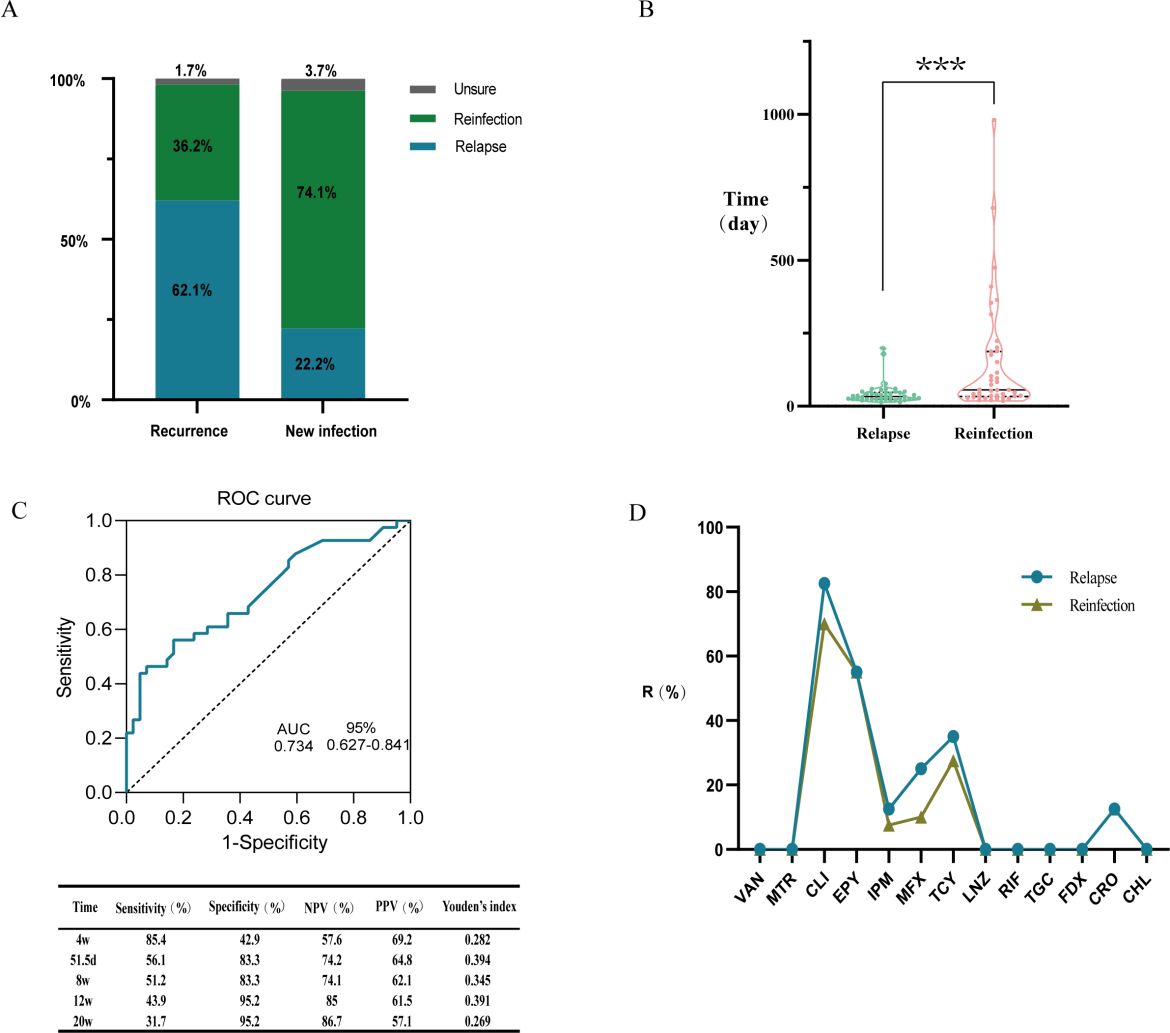
Comparison of characteristics between the relapse group and the reinfection group (Relapse: Infection with the same clonal strain before and after recurrence; Reinfection: Infection with different strains before and after recurrence); (**A**) Proportion of relapsing strains and reinfection strains among recurrent and new infections defined by an 8-week interval (%), Unsure: There are 3–10 differential SNPs between strains before and after recurrent infection. (**B**) Interval time to recurrence in the relapse group and reinfection group. (**C**) Receiver operating characteristic (ROC) curve distinguishing reactivation from reinfection by time interval and characteristics at different cutoff times. (**D**) Resistance rates (R %) to 13 antimicrobial agents in primary infection strains between the relapse group and the reinfection group. ***P < 0.001.

According to the WGS results, based on the single nucleotide polymorphism (SNP) analysis, strains with ≤2 SNPs are considered identical clones (21). Strains with 3–10 SNP differences are suspected to be the same strain, whereas those with >10 SNP differences are classified as distinct strains. Among 82 patients with recurrent CDI, 40 cases (82 strains) were classified as relapse and 40 cases (81 strains) as reinfection based on this threshold. The remaining two cases (four strains) were classified as indeterminate (with 3–10 SNPs differing between pre- and post-infection strains). According to the 8-week cutoff value for recurrence definition, 55 and 27 patients were classified as recurrence and new infection cases, respectively. Among 55 recurrent CDI cases, a total of 58 recurrence events occurred, with 36 recurrences (36/58, 62.1%) involving infection with the same clonal strain before and after. Among the 27 newly infected cases of CDI patients, six cases (6/27, 22.2%) were infected with the same clonal strain before and after (recurrence intervals of 59 days, 59 days, 60 days, 77 days, 175 days, and 199 days) (Fig. 1A).

Comparing clinical characteristics between CDI patients with relapse and patients with reinfection (Table 1), statistically significant differences were observed in relapse and reinfection rates between HA-CDI and CA-CDI patients (*P* < 0.05), with the former category exhibiting a higher relapse rate than the latter one. Additionally, the recurrence interval was significantly longer in the reinfection group than in the relapse group (Fig. 1B). No statistically significant differences were observed between the two groups in terms of sex, age, hospitalization duration, pre-diagnosis exposure factors, initial severity, treatment duration, or therapeutic agents (vancomycin or metronidazole).

**TABLE 1:** Analysis of clinical characteristics in CDI patients with infection relapse and reinfection^*a*^

Clinical characteristics		CDI patients with relapse (*n* = 40)	CDI patients with reinfection (*n* = 40)	*P* value^*b*^
Average age (years) (mean + std）		63.9 + 2.945	62.4 + 3.299	0.735
	≥65 years	17	19	0.822
	<65 years	23	21	
Sex	Male	25	25	1
	Female	15	15	
CDI Epidemiological classification (excluding nine untraceable cases)	HA-CDI	30	26	0.039*
	CA-CDI	3	12	
Exposure before initial diagnosis of CDI	Bone marrow transplant	4	2	0.675
	Antibiotic exposure during the first 8 weeks	28	22	0.168
	History of chemotherapy within the past 3 months	4	9	0.225
Initial severity of CDI onset	Severe CDI	9	4	0.225
	Non-severe CDI	31	36	
Length of hospital stay, median (IQR)		86 (33, 173）	36 (9, 142）	0.062
Recurrence interval, median (IQR)		33.5 (24, 47.5）	56 (33.5, 187.5）	0.000*
Antibiotic treatment for initial infection (vancomycin/metronidazole)	Vancomycin	19	14	0.442
	Metronidazole	7	2	
Number of days of antibiotic treatment for initial CDI infection (vancomycin/metronidazole)	<10 days	15	10	0.134
	10–14 days	9	2	
	>14 days	2	4	

^*a*^ CA-CDI, community-associated CDI; CDI, *C. difficile* infection; HA-CDI, healthcare-associated CDI; IQR, interquartile range.

^*b*^ “*” indicates *P* < 0.05.

To validate the optimal cutoff value for distinguishing relapse from reinfection in this study, a ROC curve analysis was performed based on WGS results. The AUC was 0.734 (95% CI: 0.627–0.841). Youden’s index identified 51.5 days as the optimal cutoff for differentiation; at this cutoff, sensitivity, specificity, negative predictive value, and positive predictive value were 56.1%, 83.3%, 74.2%, and 64.8%, respectively (Fig. 1C). The Hosmer–Lemeshow goodness-of-fit test indicated good model fit (*χ*² = 3.117, df = 8, *P* = 0.927). Using the 8-week cutoff to distinguish between relapse and reinfection yielded sensitivity, specificity, negative predictive value, and positive predictive value of 51.2%, 83.3%, 74.1%, and 62.1%, respectively. Youden’s index values at the 8-week, 12-week, and 20-week cutoff points were 0.345, 0.391, and 0.269, respectively.

### Multi-locus sequence typing

Through the MLST analysis, 28 ST types were identified among 167 isolates, with ST3 (21/167, 12.6%), ST2 (21/167, 12.6%), ST35 (19/167, 11.3%), ST54 (16/167, 9.6%), ST102 (16/167, 9.6%), ST42 (12/167, 7.2%), ST129 (9/167, 5.4%), and ST37 (7/167, 4.2%), with 72.5% of strains belonging to these ST types. The relapse group was dominated by ST102 (12/82, 14.6%), while the reinfection group was dominated by ST2 (13/81, 16.0%), showing increased ST diversity. HA-CDI patients were predominantly infected with ST3 (15.4%) and ST35 (12.8%), while CA-CDI patients were primarily infected with ST2 (15.6%) and ST54 (12.5%) (Table 2). No highly virulent ST1 (BI/NAP1/027) strains were detected. This study did not identify any specific ribosomal typing associated with recurrence caused by infection with the same genotype (*P* = 0.596).

**TABLE 2:** Distribution of *C. difficile* MLST^*a*^

MLST	Relapse (*N* = 82)		Reinfection (*N* = 81)		Unsure (*N* = 4)		HA-CDI	CA-CDI
	*N*	%	*N*	%	*N*	%	*N*	*N*
ST2	8	9.8%	13	16.0%	0	0%	11	5
ST3	10	12.2%	11	13.6%	0	0%	18	2
ST35	10	12.2%	9	11.1%	0	0%	15	2
ST102	12	14.6%	4	4.9%	0	0%	10	1
ST54	6	7.3%	6	7.4%	4	100%	12	4
ST42	8	9.8%	4	4.9%	0	0%	7	3
ST129	4	4.9%	5	6.2%	0	0%	8	1
ST8	4	4.9%	1	1.2%	0	0%	2	3
ST37	4	4.9%	3	3.7%	0	0%	7	0
ST5	2	2.4%	2	2.5%	0	0%	2	0
ST14	2	2.4%	3	3.7%	0	0%	4	1
ST81	2	2.4%	4	4.9%	0	0%	5	1
ST139	2	2.4%	2	2.5%	0	0%	2	2
Other ST	8	9.8%	14	17.3%	0	0%	14	7

^*a*^ Unsure: There are 3–10 differential SNPs between strains before and after recurrent infection. The statistical distribution of MLST types among HA-CDI and HA-CDI patients should exclude the 9 cases (18 isolates) that could not be traced.

### Antimicrobial susceptibility testing

Antimicrobial susceptibility testing using the agar dilution method on 167 *C. difficile* isolates revealed no resistance to vancomycin, metronidazole, linezolid, chloramphenicol, or tigecycline. Clindamycin (78.4%, 131/167) and erythromycin (58.7%, 98/167) exhibited the highest resistance rates, followed by tetracycline (29.9%, 50/167), moxifloxacin (22.8%, 38/167), ceftriaxone (14.4%, 24/167), and imipenem (9.6%, 16/167). One strain exhibited rifampicin resistance (MIC 128 mg/L), and one strain showed fidaxomicin resistance (MIC 1 mg/L) (relapse group) (0.6%, 1/167). Approximately 41.9% of *Clostridioides difficile* isolates were multidrug-resistant (70/167). In the relapse group, 45.1% were multidrug-resistant (37/82), while 38.3% in the reinfection group were multidrug-resistant (31/81) (*P* = 0.428). To investigate differences in resistance profiles between initial isolates in the relapse and reinfection groups, antimicrobial susceptibility testing revealed higher resistance rates in the relapse group compared to the reinfection group for clindamycin, imipenem, moxifloxacin, and tigecycline (Fig. 1D); however, these differences were not statistically significant (Table 3).

**TABLE 3:** Antimicrobial resistance rates and susceptibility rates for all *C. difficile* isolates, as well as for the initial infection strains in the relapse and reinfection groups^*d*^

Antimicrobial agents	MIC range (mg/L)	All (*N* = 167)			Relapse (*N* = 40)			Reinfection (*N* = 40)		
		MIC_50/90_ (mg/L)	*R* (%)	*S* (%)	MIC_50/90_ (mg/L)	*R* (%)	*S* (%)	MIC_50/90_ (mg/L)	*R* (%)	*S* (%)
Vancomycin^*a*^	0.125–32	0.5/1	0	100	0.5/1	0	100	0.5/1	0	100
Metronidazole	0.06–128	0.125/0.25	0	100	0.125/0.25	0	100	0.125/0.25	0	100
Clindamycin	0.125–32	32/32	78.4	4.8	32/32	82.5	5.0	32/32	70	0
Erythromycin	0.06–256	256/256	58.7	41.3	256/256	55	45.0	256/256	55	45.0
Imipenem	0.125–64	4/8	9.6	70.1	4/16	12.5	62.5	4/8	7.5	77.5
Moxifloxacin	0.25–32	2/16	22.8	75.5	2/16	25	75.0	1/4	10	87.5
Tetracycline	0.06–64	4/16	29.9	59.3	0.06/1	35	50.0	0.125/16	27.5	60.0
Linezolid	0.06–128	1/2	0	100	1/2	0	100	0.5/2	0	100
Rifampicin	0.06–128	0.06/0.06	0.6	99.4	0.06/0.06	0	100	0.06/0.06	0	100
Tigecycline^*b*^	0.06–64	0.06/0.5	0	100	0.06/1	0	100	0.06/0.125	0	100
Fidaxomicin^*c*^	0.015–64	0.03/0.25	0.6	99.4	0.03/0.25	0	100	0.015/0.125	0	100
Ceftriaxone	0.125–256	16/64	14.4	62.3	16/64	12.5	62.5	16/64	12.5	67.5
Chloramphenicol	0.125–128	4/8	0	99.4	4/4	0	100	4/8	0	100

^*a*^ Vancomycin breakpoint reference follows the European Committee on Antimicrobial Susceptibility Testing (EUCAST) standard (susceptible ≤2 mg/L, resistant >2 mg/L).

^*b*^ Tigecycline breakpoint reference follows the U.S. Food and Drug Administration (FDA) criteria (susceptible ≤4 mg/L, resistant ≥16 mg/L).

^*c*^ Fidaxomicin breakpoint reference follows the epidemiological cut-off values (ECOFFs) of EUCAST standards (susceptible ≤0.5 mg/L, resistant >0.5 mg/L).

^*d*^ MIC, minimum inhibitory concentration; R, resistant; S, susceptible; All, all strains; N: number of strains.

### Analysis of antibiotic resistance genes, virulence genes, and mobile genetic elements

The antibiotic resistance of *C. difficile* to various antimicrobials is associated with the carriage of corresponding resistance genes. Fifteen antibiotic resistance genes were identified across all isolated strains, primarily including *erm*B (associated with clindamycin and erythromycin resistance), the *tet* family (associated with tetracycline resistance), and *catD* (associated with chloramphenicol resistance). Additionally, *aph (2'')-If*, *marA*, *patA*, and *tet32* resistance genes were identified exclusively in a few strains from the relapse group. Nearly, all (97.8%, 91/93) *erm*B-positive strains were resistant to erythromycin; however, of the 131 clindamycin-resistant strains, only 91 (69.5%) carried the *erm*B gene, indicating the presence of alternative resistance mechanisms. The *tet*M gene was detected in 52.0% (26/50) of tetracycline-resistant isolates. The chloramphenicol-associated *catD* resistance gene was present exclusively in ST35 strains. Most *C. difficile* isolates resistant to moxifloxacin were found to have *gyrA* mutations (84.2%, 32/38). On average, strains in the relapse group contained 3.22 antibiotic resistance genes, while those in the reinfection group contained 2.90 genes, with no statistically significant difference (*P* = 0.095). Among clindamycin-resistant strains in the relapse group, ST102 (12/70, 17.1%) was the predominant *erm*B genotype, whereas ST2 (9/57, 15.8%) was predominant in the reinfection group; however, this difference was not statistically significant (*P* = 0.999).

**Fig 2:**
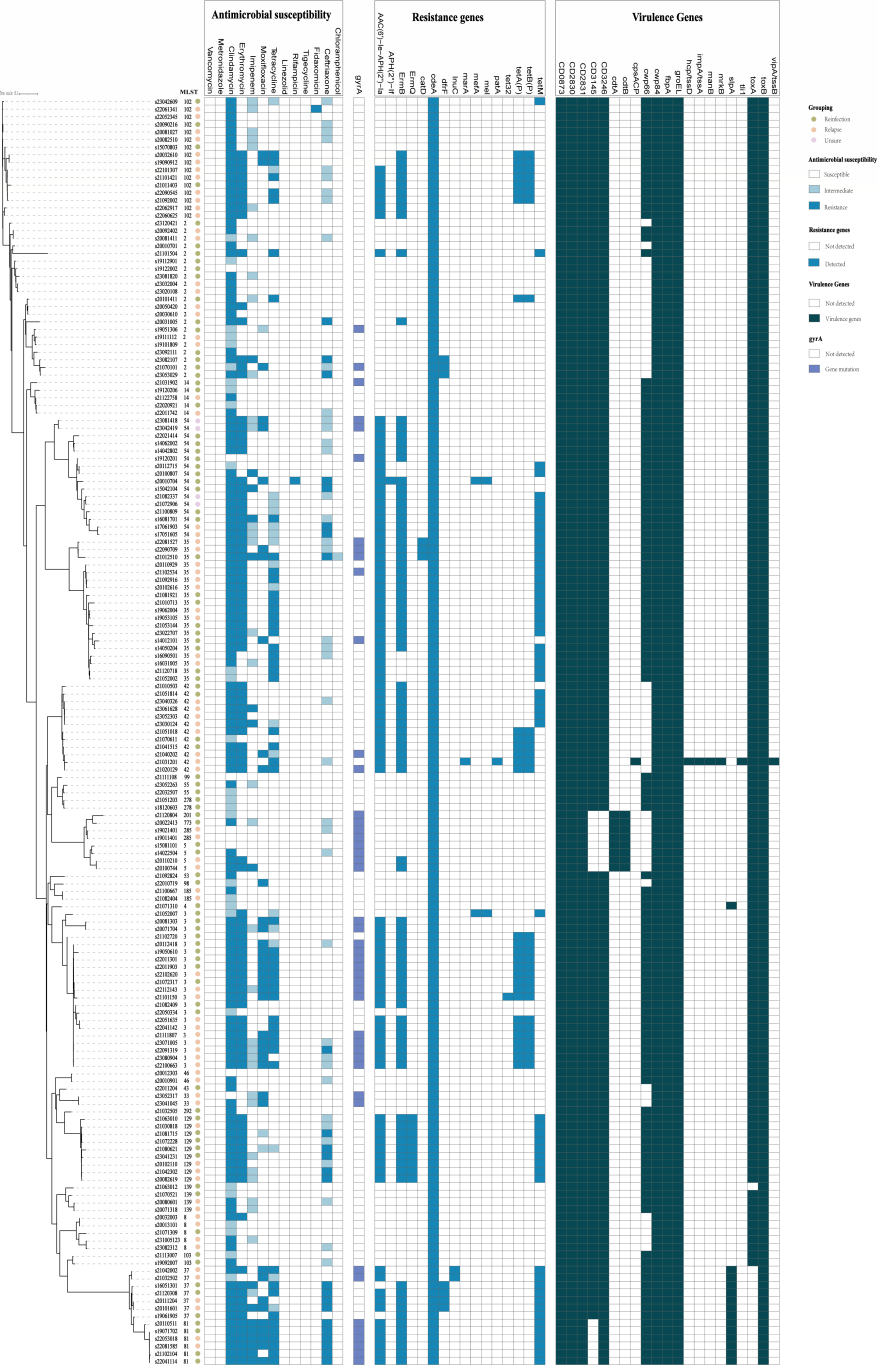
A phylogenetic tree of 167 *C. difficile* strains was constructed based on core genome single nucleotide polymorphisms (SNPs), incorporating results from multi-locus sequence typing (MLST), antimicrobial susceptibility testing, and analyses of resistance and virulence genes.

Analyses of virulence genes in the initial infection strains of the relapse group and reinfection group revealed no significant differences in virulence genes. All isolated strains carried virulence genes including *CD0873*, *CD2830*, *CD2831*, *cwp84*, *fbpA*, and *groEL*. The virulence gene *cwp66*, which encodes a cell wall protein with adhesive properties acting as a colonization factor (22), was present in most isolates (71.9%, 120/167). In the relapse group, it was predominantly found in ST102 (20.7%, 12/58), while in the reinfection group, it was predominantly carried by ST3 (18.9%, 11/58) (*P* > 0.999). The virulence genes *cpsACP*, *impA/tssA*, *manB*, *mrkB*, *tli1*, and *vipA/tssB* were detected only in a single ST42 strain, in the relapse group (Fig. 2).

The transposons carrying antibiotic resistance genes isolated from the strains in this study primarily included Tn*916* (associated with tetracycline and minocycline resistance), Tn*6189* (associated with erythromycin resistance), and Tn*4453a* (associated with the chloramphenicol resistance gene *catD*) (23). Transposons were present in 17 relapsed strains and 17 reinfected strains, with no statistically significant difference in frequency (*P* = 0.97). No specific clustering of these transposons among different strains was observed in relation to relapsed strains. The prevalence of IS elements was significantly higher in the reinfection cohort (63 strains in relapse vs 73 in reinfection, *P* = 0.0338), with a significantly greater overall genetic load and potential mutation capacity than in the relapse group (mean 11.95 in relapse vs 12.20 in reinfection, *P* = 0.0282). Differences in IS element distribution between the two groups suggest they may play distinct roles in strain adaptive evolution. However, not all insertion sequences showed substantial associations with clinical infection types, and reinfection was caused by different strains, indicating marked strain heterogeneity.

### Genome analysis of relapse and reinfection isolates

**Fig 3:**
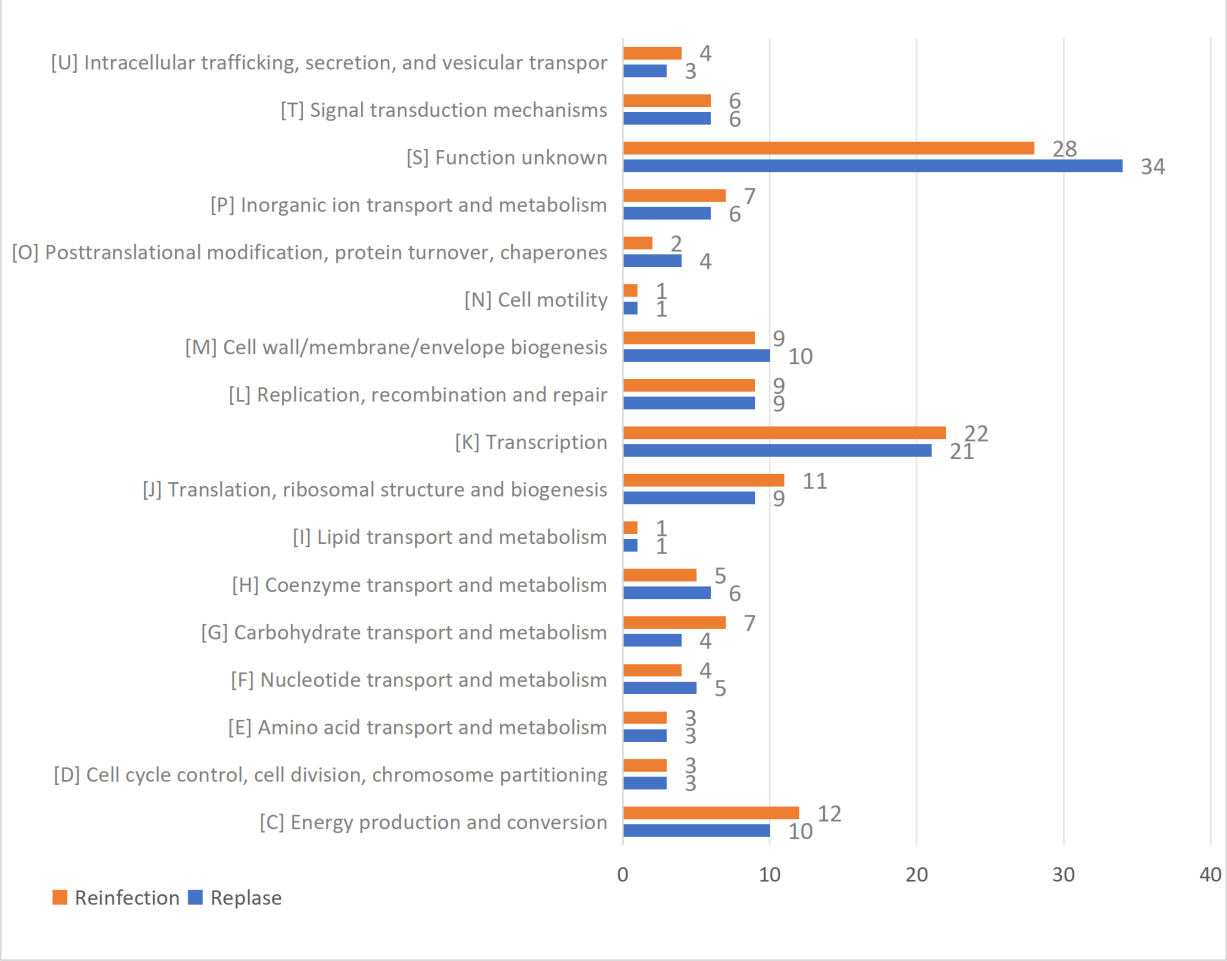
Distribution of COG functional categories of positive selection genes (*d*_N_/*d*_S_ > 1) in the core genomes of the relapsed strains and the reinfected strains.

To identify specific genes playing a key role in relapse and reinfection, a pan-genomic analysis was conducted on the initial infection strains involved in relapse and reinfection. Eleven genes showed statistically significant differences (*P* < 0.05), but these differences became nonsignificant after multiple hypothesis testing correction using the Bonferroni method. Subsequently, a *d*_N_/*d*_S_ analysis was performed on the core genomes of strains from the relapse and reinfection groups, and genes were classified into COG functional categories. Both relapse and reinfection strains exhibited a highly consistent global pattern: the number of genes undergoing purifying selection (*d*_N_/*d*_S_ < 1) significantly exceeded those under positive selection (*d*_N_/*d*_S_ > 1). The relapse strains group showed 57 genes under positive selection, while the reinfection strains group had 59. Genes under positive selection showed not only similar numbers but also highly consistent functional annotations. Most were associated with transcriptional regulatory mechanisms and energy metabolism systems, suggesting that strains may gain adaptive advantages by optimizing gene expression programs and energy utilization efficiency. Additionally, fine-tuning of cell surface structures and DNA repair mechanisms may play auxiliary roles in evading host immunity and promoting evolution (Fig. 3).

## DISCUSSION

To our knowledge, this is the first study conducted in China examining the genomic features of *C. difficile* strains causing recurrent infections. Using WGS technology, we systematically compared the genetic properties of relapsed and reinfected strains, including resistance genes, virulence genes, and MGEs, and found no significant genomic differences. Analyses of *d*_N_/*d*_S_ ratios in the core genomes of relapsed and reinfected strains revealed that most core genes underwent purifying selection in both scenarios, with only a few number of genes showing positive selection. These positively selected genes exhibited high similarity, suggesting that evolutionary drivers for relapse and reinfection strains may be similar within the core genome.

The recurrence rate of *C. difficile* infection in this study was 11%, which is lower than that reported in other European and American countries (24). This recurrence rate was based on laboratory test data and may differ from actual circumstances. Some patients were discharged after initial treatment and did not undergo laboratory diagnosis when diarrhea recurred, potentially underestimating the incidence of recurrent *C. difficile* infection. Analyses of clinical characteristics between relapsed patients and reinfected patients revealed that the former category exhibited shorter interepisode intervals compared to the latter one. Relapse primarily stems from incomplete eradication of the original bacterial strain (though reinfection with the same clonal strain from the external environment cannot be ruled out). When the intestinal environment becomes favorable, residual spores can germinate and proliferate rapidly, leading to the swift recurrence of CDI symptoms. Concurrently, healthcare-associated CDI patients exhibit higher recurrence rates than community-associated cases, a trend observed across all other countries (24). Most HA-CDI patients have underlying medical conditions and receive antibiotics more frequently, leading to slower restoration of gut microbiota balance. These factors constitute high-risk elements for recurrence (6), making HA-CDI patients more susceptible to *C. difficile* strain relapse.

The use of an 8-week cutoff point to define recurrence and new infections has certain limitations. This study found that recurrent infections involved strains of different genotypes, while some cases (22.2%) of new infections occurring beyond 8 weeks still involved strains of the same genotype, with the longest recurrence interval reaching 199 days. Clinical treatment strategies differ for relapse of the same genotype vs reinfection with a different genotype (4). Currently, clinical factors alone cannot reliably distinguish between relapse and reinfection. This study also validated the discriminatory power of 8-week, 12-week, and 20-week cutoff periods. Compared to 8-week and 20-week intervals, the 12-week cutoff demonstrated a higher Youden’s index and superior discriminatory ability. Other cohort studies have similarly employed longer cutoff periods such as 12 weeks or 20 weeks (25, 26). However, this cannot serve as a quantitative indicator for defining rCDI. Therefore, this study suggests that performing WGS analyses for rCDI, when feasible, can accurately distinguish between relapse and reinfection events, enabling more targeted therapeutic and preventive interventions.

Antibiotic resistance plays a vital role in rCDI pathogenesis (27). A trend toward higher resistance was observed in the relapse group compared to the reinfection group among the initial infecting strains. Although this difference was not statistically significant, clinicians should remain vigilant regarding this change. This finding may suggest potential differences in resistance mechanisms between the two groups of strains, potentially enabling relapse strains to colonize and survive more readily within the gut. Analyses of resistance genes and transposons encoding related resistance genes in the two groups revealed no significant differences between the two sets of strains. It is speculated that this resistance trend may be associated with other resistance mechanisms, such as biofilms. Biofilm formation is considered a key factor contributing to antibiotic resistance in *C. difficile*, typically associated with reduced drug susceptibility. This enables strains to survive under antibiotic pressure selection (28–30), leading to recurrent infections. Currently, routine antimicrobial susceptibility testing for CDI strains is not routinely performed in clinical settings. Monitoring the antimicrobial susceptibility of infecting strains may provide guidance for treating recurrent infections. Additionally, we found that the virulence gene *cwp66* was predominantly present in ST102 within the relapsed group (20.7%, 6/29). Research has demonstrated that this gene encodes the Cwp66 protein, which primarily functions in adhesion and is associated with motility and stress tolerance. It plays a crucial role in antibiotic resistance and metabolic processes, aiding survival of the bacterium in the human gut (22). This enhances colonization of the strain in recurrent patients, to some extent, partially explaining the prevalence of ST102 in relapse cases.

WGS reveals the complex and dynamic genome of *C. difficile*, enabling its rapid adaptation to environments and survival. Its core genome typically encodes proteins involved in metabolism, biosynthesis, DNA replication, transport, and cell division and is also associated with pathogenic virulence, adhesion, and antibiotic resistance (31, 32). During *C. difficile* evolution, clonal lineages undergo natural selection, with harmful mutations subject to purifying selection. The relative ratio of non-synonymous to synonymous mutations (*d*_N_/*d*_S_) within the core genome is calculated to infer genetic selection pressure (33, 34). Whether in relapsed or reinfected strains, the core genome exhibits a highly consistent evolutionary pattern characterized by intense purifying selection, indicating that the core genome is highly conserved. Studies have also confirmed that lineages diverging significantly within *C. difficile* are highly susceptible to strong purifying selection (35, 36), consistent with our findings. Furthermore, despite the high conservation of the core genome, certain genes remain under positive selection. These genes exhibit high consistency in both number and functional annotation between the relapsed and reinfected strains. This convergence suggests the presence of shared selective pressures driving adaptive evolution, possibly indicating a limited yet critical adaptive evolutionary pathway (such as key transcriptional regulators or metabolic pathways) playing a significant role in *C. difficile* recurrent infections (whether CDI patients with relapse or reinfection). Furthermore, alternative explanations warrant consideration: genes under increased positive selection may reside in accessory genomes excluded from this study. Additionally, other regulatory factors such as host gut microbiota and differences in gene expression regulation should be considered, as they may play a role in recurrent *C. difficile* infections (37–39).

Nevertheless, our study has several limitations. First, only a single strain was isolated from the stool samples of all patients in this cohort, overlooking the possibility of mixed infections. Additionally, it is difficult to distinguish whether recurrence stems from a relapse of the original strain or reinfection with the same clonal strain, which underestimates the complexity of recurrent infections. Second, some cases were lost to follow-up after discharge following the initial treatment, and information on outpatient cases could not be fully collected. Each case should be followed up on for a long time to ensure date accuracy. Additionally, the sample size was small, introducing potential biases and limitations that may restrict applicability to the current setting.

### Conclusion

To the best of our knowledge, this study is the first to investigate the genomic characteristics of recurrent *C. difficile* infection strains in mainland China. The 8-week recurrence interval definition has limitations and requires reevaluation. No significant differences were observed in the genomes of the relapsed and reinfected strains, including transposons, resistance genes, and virulence genes. Most genes in recurrent strains underwent strong purifying selection pressure targeting the core genome. Transcriptional regulatory mechanisms and energy metabolism systems in recurrent strains may play a role, albeit limited. Notably, MIC increments were observed in patients with relapse, suggesting that other potential alternative mechanisms may influence bacterial survival and play a role in different types of recurrent infections. This study has limited data; therefore, large-scale prospective cohort studies are urgently necessary to validate adaptive evolutionary pathways in recurrent strains. It is hoped that this study will serve as a reference for addressing rCDI issues in the future.

## Data Availability

The whole-genome sequences of *C. difficile* strains generated in this study have been deposited in the NCBI Sequence Read Archive (SRA) under the BioProject accession number PRJNA1418975.
